# New Updates in Adipocytes and Adipose Tissue

**DOI:** 10.3390/life16020300

**Published:** 2026-02-10

**Authors:** Endre Kristóf, Éva Csősz

**Affiliations:** 1 Laboratory of Cell Biochemistry, Department of Biochemistry and Molecular Biology, Faculty of Medicine, University of Debrecen, H-4032 Debrecen, Hungary; 2 Proteomics Core Facility, Department of Biochemistry and Molecular Biology, Faculty of Medicine, University of Debrecen, H-4032 Debrecen, Hungary

## 1. Introduction

Obesity is a major health problem in developed countries and a growing one in the developing world. It increases the risk of diabetes mellitus, heart disease, fatty liver, and certain types of cancer [1]. In Europe, approximately four million people die each year as a result of cardiovascular diseases [2]. A better understanding of the biological basis of obesity should aid its prevention and treatment. The terms obesity and overweight refer to excess in body weight relative to height. The body mass index (BMI) is well associated with measures of body fat in most individuals and it is defined as weight divided by height in meters on the second order (m^2^). Overweight is defined at 25–30 kg/m^2^ BMI while obesity is considered when the BMI of an individual exceeds 30 kg/m^2^ [1].

Body fat, the stored mass of triacylglycerol (TAG), is in the adipose tissue, which is widely distributed in the body, primarily under the skin, around vessels, and in the abdominal cavity, amounting to 11 kg in a normal average adult human [3]. According to recent evidence the fat depots are not uniform; the increase in the abdominal fat has a more pronounced effect on insulin resistance and is considered a hallmark of metabolic syndrome (MetS) [4]. Although in most of the cases the obesity is defined by increased BMI, the importance of waist circumference or waist to hip ratio (WHR) gains more and more attendance and there are recommendations to consider WHR along with BMI when defining obesity [5].

Adipose tissue, an extraordinarily flexible and heterogeneous organ, is classified into two basic types, white and brown adipose tissues (WAT and BAT), each with distinct anatomical locations, functions, and cellular compositions, which work in tandem to regulate the energy balance of the entire organism [3]. All types of adipocytes accumulate TAGs in lipid droplets. White adipocytes form a single large lipid vacuole (30–100 μm) in vivo and contain only a thin rim of cytoplasm around them. This appearance is often referred to as unilocular morphology. Contrarily, brown/beige adipocytes accumulate numerous smaller lipid droplets in a multilocular arrangement. They have a polygonal or ellipsoid shape (20–40 μm diameter) [6]. In most cases, clear anatomical borders between BAT and the surrounding WAT depots do not exist. Furthermore, it should be emphasized that 20–50% of the cells in any fat depot are not adipocytes and constitute the so-called stromal–vascular fraction. This fraction contains vascular elements, adipose tissue-derived mesenchymal stem/stromal cells (ADMSCs), fibroblasts, other progenitors, macrophages, lymphocytes, mast cells, and nerves [6,7,8].

This Special Issue focuses on metabolic changes and novel molecular pathways observed in obesity with a high emphasis on adipose tissues that can be targeted in the fight against obesity and MetS. It contains eight research articles and two reviews that revolve around the main topics of adipogenesis, inflammation and insulin resistance, or thermogenesis.

## 2. Novel Methods and Regulators of Adipogenesis

### 2.1. Application of ADMSCs in Basic Research and Clinical Practice

The composition of adipose tissue is in constant flux due to persistent low-level turnover and the replacement of adipocytes at a rate of approx. 10% per year in humans [3]. Both white and brown adipocytes are able to store and liberate TAGs and express a common set of adipogenic marker genes. They arise from ADMSCs via complex differentiation processes primarily controlled by the peroxisome proliferator–activated receptor (PPAR)-γ and members of the CCAAT/enhancer-binding protein (C/EBP) family of transcription factors [6]. Paracrine and endocrine cues regulate distinct phases of adipogenesis out of which insulin activates lipogenesis and stimulates the entire differentiation program [9].

The availability of in vivo models is restricted in several cases because of ethical or technical limitations and they cannot reproduce adipogenesis completely in humans. To overcome these limitations, several in vitro and in silico methods were designed that were comprehensively reviewed by Pamplona et al. These involve traditional 2D cell cultures, application of hormonal cocktails to induce and maintain the differentiation of adipocytes from ADMSCs or preadipocyte cell lines, assessment of the expression of key adipogenic markers and the activity of crucial metabolic pathways, and the prediction of molecular interactions. In association with other recent reviews [8,10], advances in 3D adipocyte/adipose tissue models and organ-on-a-chip systems are highlighted.

Mubtasim and Gollahon optimized the adipogenic differentiation protocol of the clonal derivative of the murine 3T3-L1 preadipocyte cell line, 3T3-L1 MBX cells. The adipogenic cocktail that resulted in the most effective differentiation was composed of 0.5 mM isobutyl-methylxanthine, 1 µM dexamethasone, 10 µg/mL insulin, and 2 µM rosiglitazone, and was incubated for 3 days with the preadipocytes. When 3T3-L1 MBX cells were differentiated in the presence of a mixture of fatty acids (FAs), hypertrophic adipocytes were developed with altered adipokine secretion, which resembles the obesogenic conditions more properly.

Lee et al. applied micro-current stimulation on 3T3-L1 preadipocytes prior to their differentiation, which inhibited insulin signaling, downregulated C/EBP-α expression, and prevented PPAR-γ nuclear translocation. This resulted in a decrease of approx. 30% in lipid accumulation in vitro. Furthermore, the beneficial effects of micro-current stimulation were validated in vivo. In the monogenic obesity model of *ob/ob* mice, weight gain, WAT expansion, and ectopic lipid deposition in the liver were partially attenuated along with downregulation of C/EBP-α in epididymal WAT.

Undifferentiated ADMSCs regulate local tissue homeostasis, e.g., promote angiogenesis, suppress pro-inflammatory signaling, and support tissue repair, via released factors or cell–cell interactions [11]. However, in individuals with obesity, these beneficial effects are often compromised because of the pro-inflammatory and hypoxic milieu in hypertrophic WAT, limiting the therapeutic potential of ADMSCs in regenerative medicine. The comprehensive review of Alma et al. focuses on this issue especially by highlighting ADMSC-based therapeutic interventions to aid the healing of chronic ulcers, particularly in patients with obesity in which vascular insufficiency, chronic inflammation, and metabolic dysfunction disrupt the physiological pathways of tissue repair.

### 2.2. Novel Regulators of Adipogenesis: Focus on Polyunsaturated Fatty Acids (PUFAs) and Prostanoids

The prostanoid family of eicosanoid lipid mediators are synthesized and released by WAT and BAT. Prostaglandins (PGs) exert important regulatory functions with regard to inflammation, lipolysis, adipogenesis, thermogenesis, browning, and vascular tone in adipose tissues as autocrine/paracrine factors. Prostacyclin (PGI_2_) and the PGJ_2_ derivatives, 15-deoxy-Δ^12,14^-PGJ_2_ and Δ^12^-PGJ_2_, promote adipocyte differentiation. PGF_2α_ inhibits adipogenesis, while the effects of PGE_2_ and its metabolites are context-dependent in this respect [12]. In this Special Issue, Nartey et al. published three follow-up research articles in which the differentiation of 3T3-L1 preadipocytes was inhibited in the presence of excess PGD_2_, arachidonic acid (AA), or eicosapentaenoic acid (EPA).

When PGD_2_ or its chemically stable analog, 11-deoxy-11-methylene-PGD_2_, were administered during the first two days of differentiation, the mature 3T3-L1-derived adipocytes accumulated less TAG and expressed key adipogenic marker and D-prostanoid receptor (DP) 1 and 2 genes at a lower extent. The anti-adipogenic effects of PGD_2_ and its analog were attenuated by a DP2 and a PGI_2_ receptor agonist, respectively.

As a next step, preadipocytes were differentiated in the presence of AA or its precursor, linoleic acid (LA), in a similar experimental setup. LA is the most abundant PUFA in the Western diet and accounts for 85–90% of dietary ω-6 PUFAs. Its richest dietary sources are vegetable oils and the seeds of several plants. LA and AA are also present in significant amounts in animal products such as chicken eggs or lard [13]. AA, but not LA treatment, decreased TAG accumulation and downregulated the mRNA expression of adipogenic markers, which had effects that could not be prevented when pro-adipogenic PGs were co-administered. The altered secretion of distinct PG derivatives as a result of AA treatment might at least partially underlie the observed suppression of in vitro adipogenesis.

Finally, the effects of other FAs were investigated during the differentiation of the most commonly used murine preadipocyte cell line. Out of the five applied FAs, only EPA administration resulted in similar anti-adipogenic effects as PGD_2_ or AA which was attenuated by Δ^12^-PGJ_2_. Many animals, as well as humans, can synthesize critical ω-3 and ω-6 PUFAs, such as AA, EPA, and docosahexaenoic acid, from the essential FAs, LA, or α-linolenic acid, although the efficiency of this synthesis can vary. EPA, docosapentaenoic acid, and docosahexaenoic acid are found in fish, especially in so-called “oily” fish products (tuna, salmon, mackerel, herring, and sardine) [14]. EPA increased cyclooxygenase-2 expression at mRNA level and protein kinase A activity of mature 3T3-L1-derived adipocytes, which could at least partially explain its adipogenesis-suppressing effects. Further research is required to validate these findings in primary adipocyte and in vivo models and to explore the detailed molecular mechanisms of how distinct FAs or PGs regulate adipocyte differentiation.

## 3. Inflammation and Insulin Resistance: The Hallmarks of MetS

Adipocyte hypertrophy results in hypoxia because of the massively enlarged cell size, which upregulates the expression of pro-fibrotic genes and leads to WAT fibrosis. Moreover, hypoxia can result in necrotic cell death and subsequent recruitment of immune cells. The released pro-inflammatory cytokines, such as tumor necrosis factor (TNF)-α and interleukin-6, maintain a chronic low-grade inflammation contributing to earlier onset of MetS [15].

Gutiérrez-Rojas et al. modeled inflammation by a single bolus of TNF-α administration to mature 3T3-L1-derived adipocytes, which resulted in the downregulation of four orphan G protein-coupled receptor (GPR) encoding genes, *Gpr26*, *Gpr39*, *Gpr82*, and *Gpr6*. Glycine treatment alone exerted anti-inflammatory effects along with the upregulation of *Gpr21*, *Gpr26*, *Gpr82*, and *Gpr6*. In addition, pre-treatment with glycine prevented the TNF-α-induced in vitro inflammatory response as well as *Gpr82* depletion.

The diagnosis of MetS requires the presence of three out of the five listed criteria at the same time: visceral adiposity, increased fasting blood glucose level, high blood pressure, elevated plasma TAG level, or decreased plasma levels of high-density lipoprotein [16]. Insulin resistance, which impairs glucose uptake and utilization in liver, muscle, and adipose tissue, is a strong predictor of type 2 diabetes mellitus [17]. Kang et al. observed that mulberry leaf extract and its bioactive component, 1-deoxynojirimycin, decreased total body weight, fasting blood glucose, serum TAG, total cholesterol, and insulin levels, and improved glucose tolerance in the monogenic obesity model of *db/db* mice. Mechanistically, the *per os* treatments increased PPAR-γ, PPAR-γ coactivator-1α, glucose transporter type 4, and insulin receptor substrate 1 protein expression, phosphorylation of critical elements of insulin signaling cascade, glycogen storage, and fiber size in gastrocnemius muscle.

## 4. BAT Thermogenesis—Can We Burn the Excess Energy in the Presence of Peripheral Serotonin?

The possibility that the heat production of BAT might combat metabolic disturbances, e.g., weight gain, was initially addressed in 1979, when the first induced thermogenic process, diet-induced thermogenesis (thermogenic capacity to combust excess energy in the diet), was described. However, it had been generally believed for decades that BAT disappears in childhood or adolescence and therefore lacks significance in human adults. This dogma was opposed approx. two decades ago when several metabolically active BAT depots were found in the adult human body, mostly enriched in the supraclavicular, deep cervical, and paravertebral regions, especially when the individuals were exposed to cold [18].

Central serotonin (5HT) is known to regulate energy balance by suppressing appetite and increasing BAT thermogenesis [19]. These actions were exploited by appetite-suppressing drugs, e.g., fenfluramine or sibutramine, in the treatment of obesity; however, they were later withdrawn because of frequent cardiovascular side effects [20]. In contrast, peripheral 5HT has an opposing effect. In association with previous findings applying murine models [21,22,23], Kesić et al. found that male rats with low peripheral 5HT levels had higher interscapular BAT mass, upregulated levels of thermogenic markers, improved cold tolerance, and lower body weight, especially when the animals were exposed to cold or a β3-adrenergic stimulus. This observation can be translated to human (patho)physiology. An atypical antipsychotic drug, clozapine, which can antagonize several 5HT receptors, reprogrammed the gene expression pattern of differentiating human ADMSC-derived and Simpson–Golabi–Behmel syndrome adipocytes in vitro, leading to an elevated expression of the browning marker uncoupling protein 1, more and smaller lipid droplets, and more mitochondrial DNA than in the untreated adipocytes [24].

## 5. Conclusions

Obesity is one of the major risk factors of insulin resistance, MetS, coronary heart disease, and cancer, which are all leading causes of mortality today. The combined prevalence of overweight and obesity has increased continuously up to 40–50% in most developed countries. Although incretin-based pharmacotherapy showed great efficacy recently, additional therapeutic approaches are needed to be developed, which is aided by the application of well optimized canonical and novel methods that model adipogenesis. Adipose tissue is a complex organ with profound effects on the homeostasis of the entire body. ADMSCs serve not only as progenitors of adipogenesis but also hold therapeutic potential in regenerative medicine. White adipocytes function as long-term energy storage by accumulating a single large lipid vacuole and produce metabolites, signaling lipids, such as PGs, and secrete protein factors (adipokines). Brown and beige adipocytes accumulate numerous small lipid droplets in a multilocular arrangement, contain a large amount of mitochondria-rich cytoplasm, and convert glucose and fatty acids into heat. This thermogenesis process is regulated by several factors such as peripheral 5HT. The production of adipose tissue-derived factors changes under different nutritional and pathological conditions that contribute to the pathophysiology of several comorbidities. The published articles in the Special Issue entitled ‘New Updates in Adipocytes and Adipose Tissue’ elucidate important novel aspects in adipose tissue biology and aim to understand the molecular background of adipogenesis, insulin resistance, or thermogenesis.

## Data Availability

Not applicable.

## Abbreviations

The following abbreviations are used in this manuscript:

5HT	Serotonin
AA	Arachidonic acid
ADMSC	Adipose tissue-derived mesenchymal stem/stromal cells
BAT	Brown adipose tissue
BMI	Body mass index
C/EBP	CCAAT/enhancer-binding protein
DP	D-prostanoid receptor
EPA	Eicosapentaenoic acid
FA	Fatty acid
GPR	G protein-coupled receptor
LA	Linoleic acid
MetS	Metabolic syndrome
PG	Prostaglandin
PPAR	Peroxisome proliferator–activated receptor
PUFA	Polyunsaturated fatty acid
TAG	Triacylglycerol
TNF	Tumor necrosis factor
WAT	White adipose tissue
WHR	Waist to hip ratio
